# Metastasis of lower gingival squamous cell carcinoma to buccinator lymph node: case report and review of the literature

**DOI:** 10.1186/s12957-019-1559-y

**Published:** 2019-01-10

**Authors:** Kaho Takada, Takeshi Kuroshima, Hiroaki Shimamoto, Toshimitsu Ohsako, Kou Kayamori, Tohru Ikeda, Hiroyuki Harada

**Affiliations:** 1 Department of Oral and Maxillofacial Surgery, Graduate School of Medical and Dental Sciences, Tokyo Medical and Dental University, 1-5-45 Yushima, Bunkyo-ku, Tokyo, 113-8549 Japan; 2 Department of Oral Pathology, Graduate School of Medical and Dental Sciences, Tokyo Medical and Dental University, 1-5-45 Yushima, Bunkyo-ku, Tokyo, 113-8549 Japan

**Keywords:** Buccinator lymph nodes, Facial lymph nodes, Metastasis, Oral cancer, Squamous cell carcinoma

## Abstract

**Background:**

Metastasis of oral cancer to the buccinator lymph nodes (BN) is uncommon. The antegrade lymphatic flow in patients with normal anatomy and physiology makes metastasis of lower gingival cancer to BN unlikely.

**Case presentation:**

A 67-year-old woman presented with a 46 × 25-mm tumor on her lower gingiva, along with metastatic foci in BN and cervical lymph nodes. After neoadjuvant chemotherapy, she underwent radical resection of the primary tumor and BN, along with neck dissection. Following surgery, she received adjuvant chemoradiotherapy. Two years after treatment, there has been no evidence of tumor recurrence or metastasis.

**Conclusion:**

This is the first report of lower gingival squamous cell carcinoma with metastasis to BN. Metastasis to BN from lower gingival cancer is very rare but should be considered in patients with locally advanced tumors or tumors that metastasize to the submandibular node.

## Background

Metastasis to the lymph nodes is the most prognostic factor in patients with oral cancer. Primary cancers in the oral region frequently metastasize to level I–III nodes, whereas metastasis of oral cancer to facial lymph nodes (FN) is rare [1, 2]. FN have been subcategorized as malar, infraorbital, buccinator, and mandibular lymph nodes [3]. Buccinator lymph nodes (BN) are present in the buccinator space along the branches of the facial vessels [4]. Metastasis of oral cancer to BN is uncommon. This report describes a patient with metastasis of lower gingival cancer to BN.

## Case presentation

**Fig. 1:**
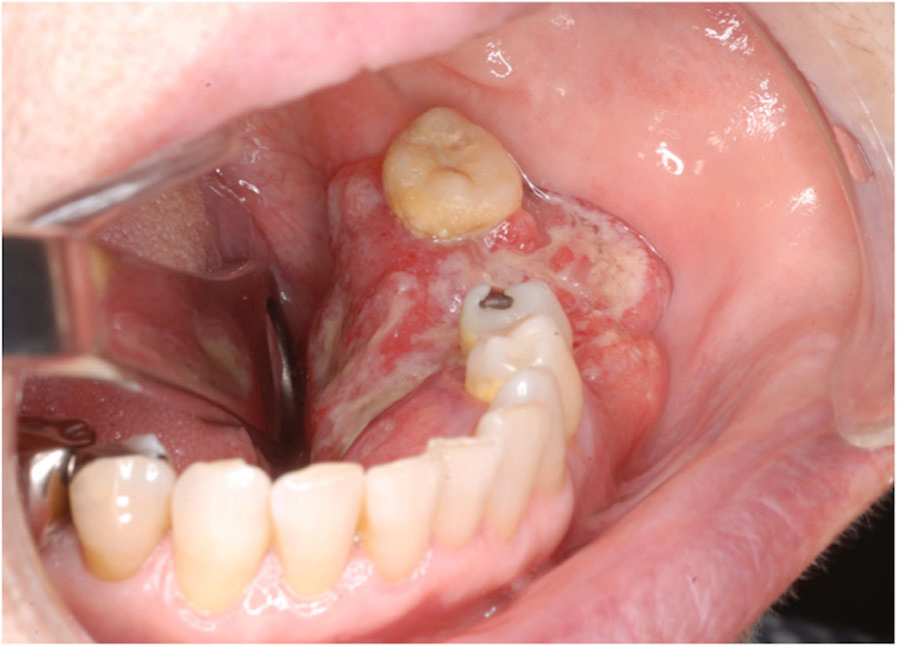
Intra-oral findings at first examination. A tumor was located in the lower gingiva on the left side

In May 2016, a 67-year-old woman came primarily to our hospital for a consultation about painless mass of the left lower gingiva. Intra-oral examination showed a 46 × 25-mm tumor with induration on the left lower gingiva (Fig. 1). A submucosal mass, independent of the gingival tumor, was palpable in the left buccal region. Several cervical lymph nodes on the left side were also palpable. Pathological examination of a biopsy sample taken from the gingival tumor revealed a well-differentiated squamous cell carcinoma.

**Fig. 2:**
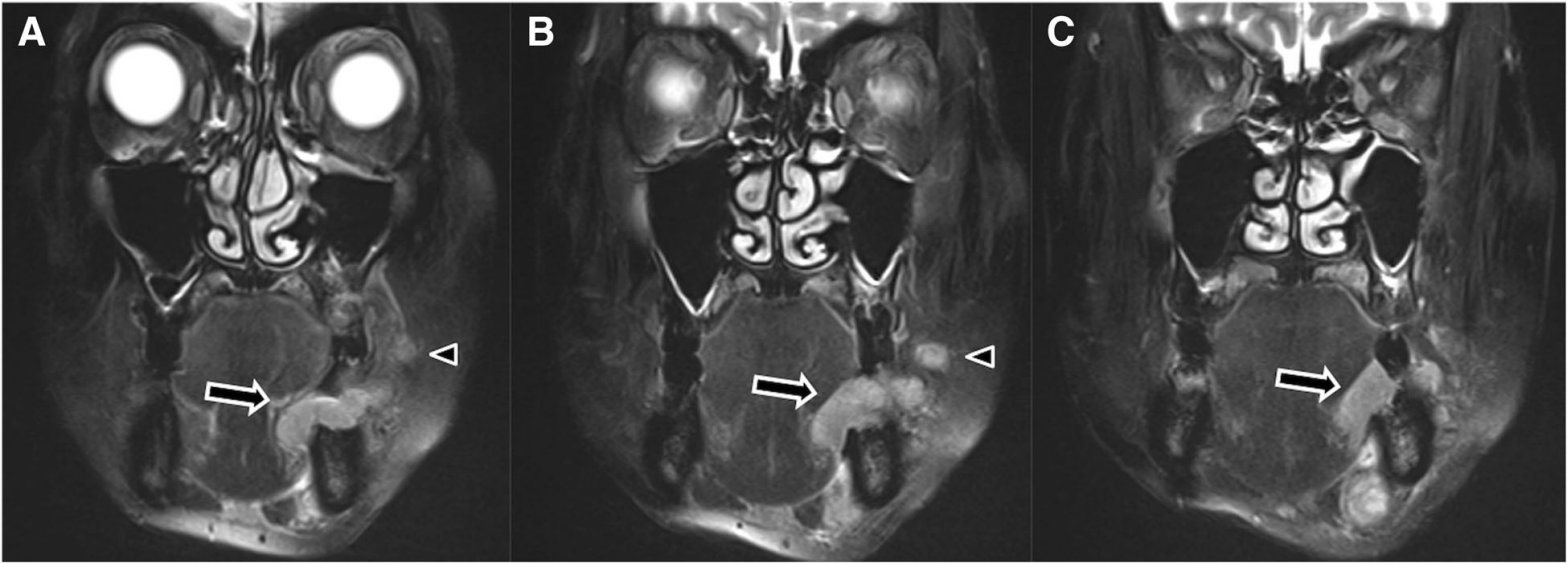
**a**–**c** Coronal fat-suppressed T2-weighted MR image showing the primary tumor (arrow) on the left side of the lower gingiva, independent of BN metastasis (arrowhead), which is embedded in the cephalad position of the primary tumor

**Fig. 3:**
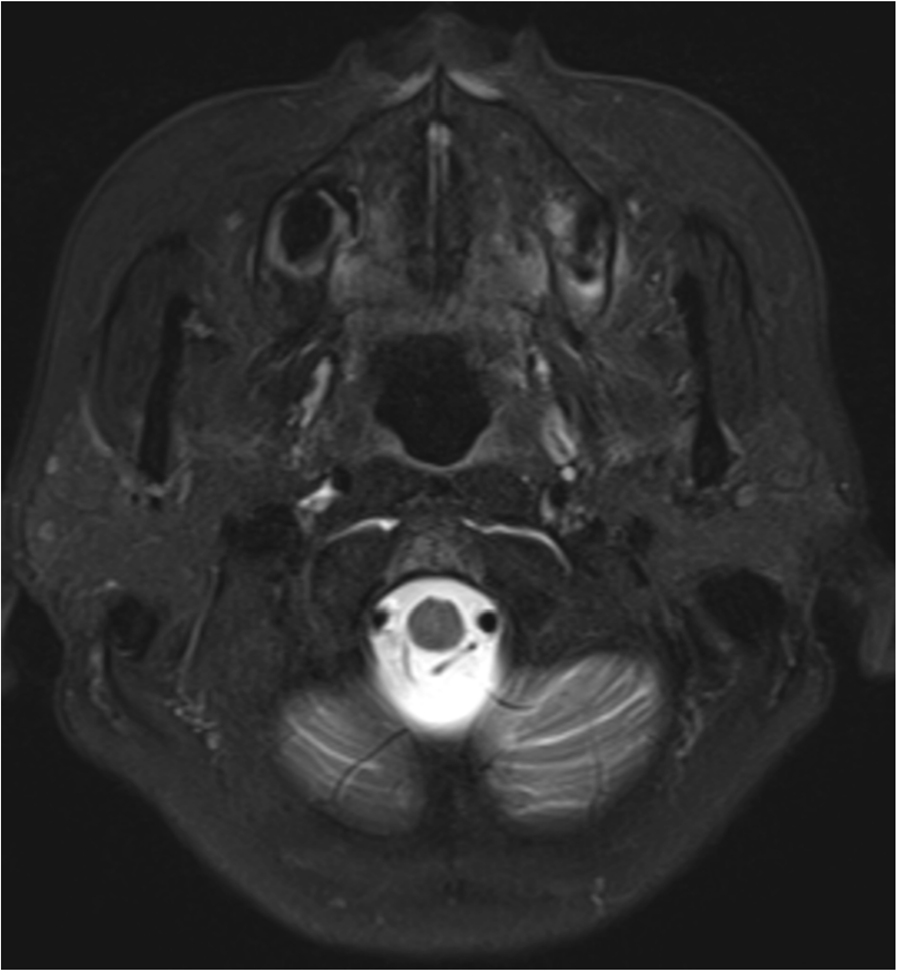
Axial fat-suppressed T2-weighted MR image showing that the primary tumor is not detected in the mandibular ramus and pterygoid region

A computed tomography (CT) scan with contrast showed a large gingival tumor, with destruction of the adjacent mandibular bone, and four metastatic left-cervical lymph nodes that were markedly enlarged, non-homogeneously enhanced, and partially necrotic. These lymph nodes included two left submandibular and two left upper jugular nodes. CT imaging showed no metastases to the lungs. Magnetic resonance imaging (MRI) showed a large primary tumor on the left side, with its epicenter located in the lower gingiva. The tumor appeared to extend into the sublingual space medially and into the buccinator muscle laterally. A non-homogeneously enhanced mass was identified in the buccinator space along the facial vessels, anterior to the anterior edge of the masseter muscle, and lateral to the buccinator muscle (Fig. 2). This mass lay on the cranial side of the primary tumor. The mandibular ramus and pterygoid region that are on the cranial side of BN were not invaded by primary tumor (Fig. 3). Moreover, T1-weighted MRI showed a thin layer with high signal, indicative of fatty tissue, between this mass and the primary tumor, indicating that the mass was independent of the primary tumor. Based on its anatomic location, the mass appeared to be metastatic disease to BN. Greyscale sonogram showed some metastatic cervical lymph nodes on the left, and metastatic BN. These cervical lymph nodes were markedly enlarged, round in shape, heterogenous hypoechoic, and without an echogenic hilus. Metastatic BN was round in shape, hypoechoic, with well-defined borders, and without an echogenic hilus.

The tumor was diagnosed as a cT4aN2bM0 squamous cell carcinoma of the lower gingiva. The patient received neoadjuvant chemotherapy, consisting of docetaxel 60–70 mg/m^2^ and cisplatin 70 mg/m^2^ on day 1, and 5-fluorouracil 700 mg/m^2^/day 96 h continuous infusion. Gross examination after two cycles of chemotherapy showed marked shrinkage of the primary tumor. A slight reduction in BN size was observed. According to the Response Evaluation Criteria in Solid Tumors (RECIST) guidelines, version 1.1 [5], this patient showed a partial response to treatment.

**Fig. 4:**
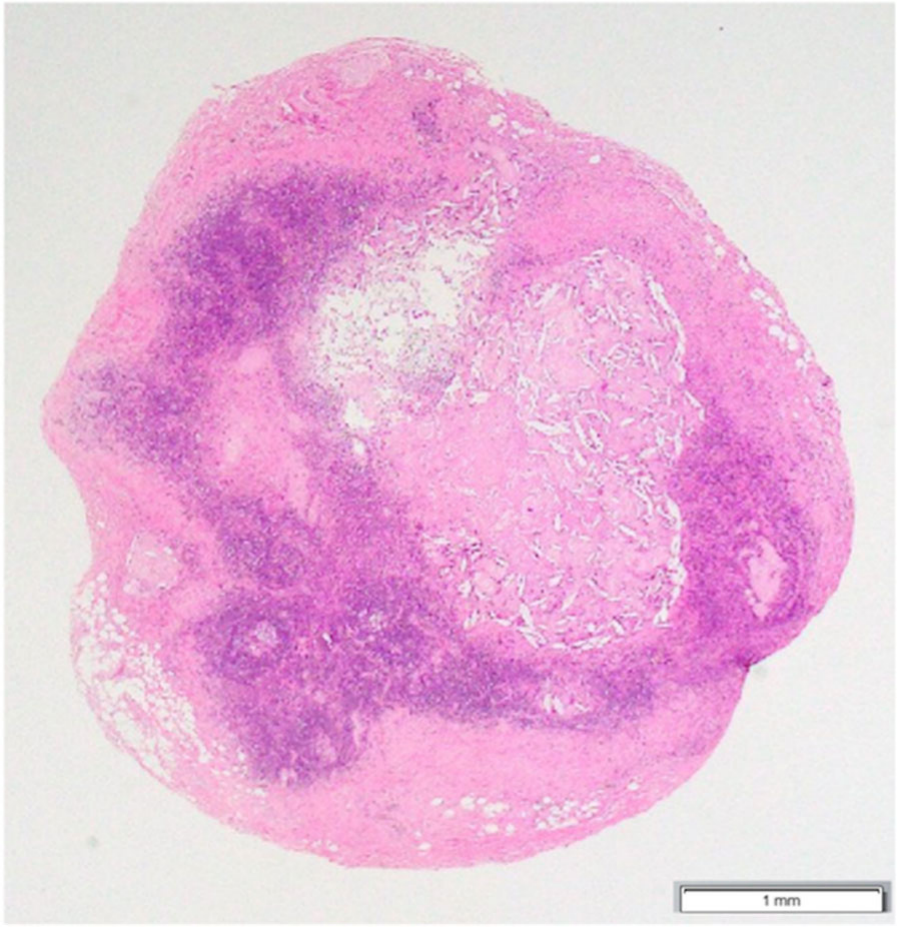
HE-stained images of the metastatic BN. The normal structure of lymph node is mainly replaced by necrotic tissue (original magnification × 20). HE, hematoxylin and eosin

Three weeks after the end of neoadjuvant chemotherapy, the patient underwent surgery, consisting of suprahyoid neck dissection (levels I–II) on the right side, classical radical neck dissection (levels I–V) on the left side, segmental mandibulectomy, and oromandibular reconstruction with a scapular osteocutaneous flap. The primary tumor and buccinator space including BN were dissected in continuity with neck dissection. Histopathological examination of the segmental mandibulectomy specimens showed that the alveolar bone and part of the bone trabeculae of the mandible had been resorbed and replaced by fibrous connective tissue. This tissue contained a few nests of squamous cell carcinoma, composed mainly of necrotic tissue with a small number of viable cancer cells and remnants of keratin pearls. The surgical margins were free from tumor. Metastatic disease was detected in five cervical lymph nodes, including one left submandibular aggregated-node, three left upper jugular nodes, and one left middle jugular node. No metastatic nodes revealed extra-nodal extension. Metastasis to BN was also present (Fig. 4). These metastatic regions contained few viable cancer cells and consisted primarily of necrotic tissue.

Following surgery, the patient was treated with adjuvant radiotherapy (50 Gy/25 fractions) with concurrent oral chemotherapy (S-1, 100 mg/day for 5 days per week for 5 weeks) [6]. Two years later, there has been no evidence of tumor recurrence or metastasis.

## Discussion

FN are inconsistently observed and frequently absent. When present, these nodes are located in the subcutaneous space along the branches of the facial vessels [4]. FN have been subcategorized into four groups: malar, infraorbital, buccinator, and mandibular lymph nodes. Among them, BN are located in the buccinator muscle and/or the fat of the buccinator space [3]. Most of the afferent drainage of BN is from the skin and subcutaneous tissues of the upper and lower eyelids, nose, and cheek. Moreover, the lymphatics of the perilabial and buccal mucosa drain directly into these nodes [7]. The efferent drainage of BN is to the submandibular nodes [3, 4].

Metastasis to BN from oral cancer is uncommon. To our knowledge, there have only been 12 oral cancer patients with metastasis to BN reported in the literature (Table 1) [3, 8–10]. The primary sites of 10 of these tumors were the maxilla and buccal mucosa, including one in the maxillary alveolar, three in the upper gingiva, and six in the buccal mucosa [3, 8–10]. Squamous cell carcinoma was the most common pathologic type, being present in eight (67%) of these 12 patients [3, 8–10]. As far as the authors are aware, to date, no patient has been described with lower gingival squamous cell carcinoma and metastasis to BN.

**Table 1:** Patients with metastasis to BN from oral cancer

Patient	Reference	Year of publication	Age/gender	Site of primary tumor	Histology	Treatment	Outcome		Prognosis^‡^
							Metastasis to BN	Primary tumor	
1	Tart	1993	ND	Buccal mucosa	Adenocarcinoma	ND	ND	Controlled	NED, 10 m
2	Tart	1993	ND	Alveolar (upper or lower were not described)	Carcinoma	ND	ND	Uncontrolled	LTF
3	Tart	1993	ND	Retromolar trigone	ND	ND	ND	Controlled	DOD
4	Tart	1993	ND	Buccal mucosa	SCC	ND	ND	Controlled	AWD, 3 m
5	Miyazaki	1999	70/M	Maxillary alveolar process	Plasmacytoma	S+RT	Controlled	Controlled	NED, 18 m
6	Kimura	2000	84/F	Buccal mucosa	SCC	Palliative	Uncontrolled	Uncontrolled	DOD, 11 m
7	Kimura	2000	53/M	Buccal mucosa	SCC	S+RT	Controlled	Controlled	NED
8	Kimura	2000	57/M	Upper gingiva	SCC	S	Controlled	Controlled	NED
9	Kimura	2000	78/M	Upper gingiva	SCC	RT	Uncontrolled	Uncontrolled	DOD, 6 m
10	Kimura	2000	75/M	Upper gingiva	SCC	S	Uncontrolled	Uncontrolled	DOD, 10 m
11	Maruoka	2005	79/M	Buccal mucosa	SCC	S^†^+RT	Controlled	Controlled	DOD, 8 y
12	Maruoka	2005	80/M	Buccal mucosa	SCC	S	Controlled	Controlled	DOD, 11 m
13	Present case	2018	67/F	Lower gingiva	SCC	CT+S+CRT	Controlled	Controlled	NED, 2 y

*Abbreviations*: *BN* buccinator lymph node, *M* male, *F* female, *SCC* squamous cell carcinoma, *ND* not described, *S* surgery, *RT* radiotherapy, *CT* chemotherapy, *CRT* chemo-radiotherapy, *AWD* alive with disease, *DOD* death from disease, *LTF* lost to follow-up, *NED* no evidence of disease

^†^Excisional biopsy of buccinator lymph node

^‡^Numbers are length of follow-up in years (y) and months (m)

BN are embedded in the buccinator space on a line running from the angle of the mouth to the inferior part of the lobule of the ear [11], that is, upstream of the lower gingiva in the lymphatic pathway. Based on normal antegrade lymphatic flow, BN are not usually sentinel lymph nodes from primary lower gingival cancer. The mechanisms of the metastasis to BN from lower gingival cancer can be considered as follows: (1) locally advanced cancer of lower gingiva reaches the adjacent buccal mucosa. Cancer cells infiltrate to the buccinator muscle and buccinator space, leading to the extension of the cephalad over the position of BN. (2) Lower gingival cancer initially spreads to neck lymph nodes, in particular to the submandibular nodes, which receive the efferent drainage of BN. Tumor spread can result in the obstruction of adjacent lymphatic vessels, blocking antegrade lymphatic flow. This would account for retrograde lymphatic flow and unexpected dissemination of cancer cells [12–14]. (3) There is a direct and retrograde pathway from lower gingiva to BN in normal physiological state, but this seems unlikely. The primary tumor of the present patient was independent of BN and located in the caudal position of BN. The tumor invaded the adjacent buccinator muscle and gave rise to large metastases to the submandibular lymph nodes. These findings may support the above hypothesis.

For radical treatment, metastasis to BN from oral cancer should be treated by surgery as should metastasis to cervical lymph node. When anatomically possible, metastatic BN should be dissected with oncological safety margins, along with dissection of the primary lesion and clinically positive neck. To remove all of these lesions, we performed en bloc resection in the present patient.

Metastasis of oral squamous cell carcinoma (OSCC) to FN has been reported to indicate advanced disease [1, 13] and to be a risk factor for local recurrence and poor prognosis [1, 2]. A study comparing outcomes in patients with OSCC found that the 5-year local control rates were 55.7% in patients with and 72% in patients without metastasis to FN (*P* < 0.001) and that the 5-year disease-specific survival rates were 43% and 57.4%, respectively (*P* < 0.001) [2]. Moreover, analysis of patients with metastases to FN who had N0–1 disease, negative surgical margins, and no extracapsular spread found that 5-year disease-specific survival rates were higher in those who received surgery followed by postoperative radiotherapy than those who received surgery alone (67.8% vs. 30.7%, *P* = 0.037) [2].

The standard strategy for advanced OSCC comprises a multidisciplinary approach including radical surgery and postoperative radiotherapy with or without systemic chemotherapy. However, the present patient received neoadjuvant chemotherapy with docetaxel-cisplatin-5-fluorouracil [15, 16] because of the waiting time for surgery [17]. Owing to the locally advanced tumor, postoperative radiotherapy concurrent with platinum-based chemotherapy was planned for the present patient. However, postoperative radiotherapy with concurrent oral chemotherapy (S-1) was performed because the patient refused use of more intravenous anti-cancer agents. S-1 is derived from 5-FU and consists of tegafur, gimeracil, and oteracil potassium [18]. It has been reported that S-1 has higher anti-tumor activity, lower side effects, and good biological availability compared with 5-FU [19, 20]. A preclinical study suggested that S-1 enhanced sensitivity to radiotherapy [21]. In clinical settings, moreover, the feasibility and efficacy of radiotherapy concurrent with S-1 were demonstrated in some cancers including oral [6], esophageal [22], and gastric cancer [23]. For these reasons, we selected S-1 as a substitute.

Of seven patients with OSCC and metastasis to BN, including the present patient, the three who received multimodal treatment, consisting of surgery and radiotherapy with or without chemotherapy, showed control of the primary tumor and BN lesion (Table 1) [3, 8, 9]. These results show that multimodal treatment might enhance local control and improve prognosis in OSCC patients with metastasis to BN.

## Conclusion

Metastasis to BN from lower gingival cancer is very rare but should be considered in patients with locally advanced tumors or tumors that metastasize to the submandibular node.

## Abbreviations

BN: Buccinator lymph nodes
CT: Computed tomography
FN: Facial lymph nodes
MRI: Magnetic resonance imaging
OSCC: Oral squamous cell carcinoma
RECIST: Response Evaluation Criteria in Solid Tumors
